# Could there be an experimental way to link consciousness and quantum computations of brain microtubules?

**DOI:** 10.3389/fnins.2024.1430432

**Published:** 2024-06-24

**Authors:** Jesús Avila, Jesús Marco, Germán Plascencia-Villa, Vladan P. Bajic, George Perry

**Affiliations:** ^1^Centro de Biología Molecular Severo Ochoa (CSIC-UAM), Madrid, Spain; ^2^Instituto de Física de Cantabria (CSIC-UC), Santander, Spain; ^3^Department of Neuroscience, Developmental and Regenerative Biology, The University of Texas at San Antonio, San Antonio, TX, United States; ^4^Laboratory for Radiobiology and Molecular Genetics, Department of Health and Environment, “VINČA” Institute of Nuclear Sciences-National Institute of the Republic of Serbia, University of Belgrade, Belgrade, Serbia

**Keywords:** consciousness, brain microtubules, quantum computations, subatomic particles, entangled stage

## Introduction

Using mathematical analyses, Penrose proposed a relation between consciousness moments and quantum computations occurring in the microtubules (MTs) of the brain. It was suggested that there is a link between brain MTs and consciousness via the entangled stage of delocalized π electrons present in the brain MTs (Hameroff and Penrose, 2014). Then, we will attempt to comment on this hypothesis step by step, looking for a possible future experimental approach that probes the hypothesis.

## Brain microtubules

We should indicate that MT assembly–disassembly dynamics requires the binding of tubulin (the main component of MTs) to GTP and the hydrolysis of this Guanosine triphsophate (GTP) to Guanosine diphosphate (GDP) (for a review, see, for example Avila, 1990; Beckett and Voth, 2023).

Brain tubulin contains a specific β-subunit isotype, which is almost exclusively present in the neurons of chordates (Sullivan and Cleveland, 1984). In addition, there is specific post-translational phosphorylation of that neuronal β subunit, whereas no such modification was found in other β-tubulin isotypes (Diaz-Nido et al., 1990).

MTs, composed of tubulin, are very abundant in the brain. By measuring tubulin levels in the cytosol of different porcine organs, including the brain, using a sensitive radioimmunoassay, it was found that tubulin accounts for 20 ± 5% of the total soluble proteins from the porcine brain (Hiller and Weber, 1978; Diez et al., 1984). Remarkably, the amount of tubulin found in peripheral tissues is ~10 to 20 times lower than the amount found for tubulin in the brain.

**Figure 1:**
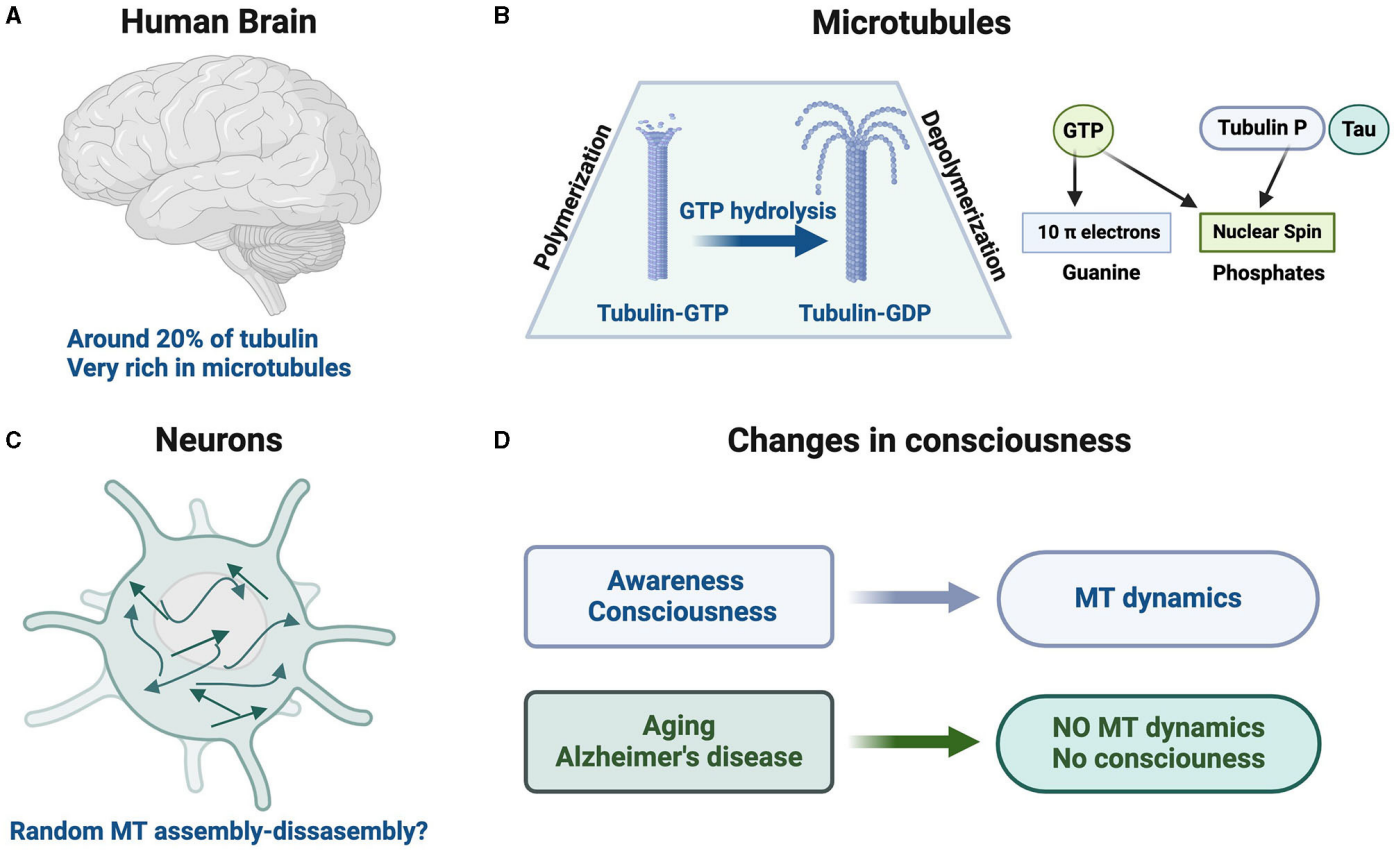
Brain microtubule dynamics, Tubulin GTP/GDP, and consciousness. **(A)** Tubulin accounts for ~20% of the total soluble brain protein. **(B)** Brain tubulin–GTP can assemble into microtubules. Upon GTP hydrolysis, brain tubulin–GDP depolymerizes from microtubules. We should indicate the presence of 10 π electrons in the guanine of GTP/GDP and the presence of nuclear spin in the phosphor of GTP/GDP. **(C)** There is a very dynamic microtubule assembly/disassembly in which microtubules can nucleate randomly through the whole neuron (Stiess et al., 2010), yielding different neuron morphologies. The role of subatomic phenomena in that process, like changes in the nuclear spin of the phosphorus present in GTP/GDP bound to tubulin, is unknown but should be analyzed if it is possible. **(D)** Proposed association of consciousness with microtubule (MT) dynamics.

Furthermore, brain MTs contain several MT-associated proteins (MAPs) that stabilize those polymers, including the tau protein (Avila, 1990). There are three specific features for brain MTs that distinguish them from MTs from other sources: (a) they are present in a higher relative amount, (b) they can nucleate in a non-centrosome/basal body-directed way, and (c) they could favor the formation of subcellular neuronal structures, such as dendrites (Figure 1).

A huge amount of tubulin is assembled into MTs from centrosomal- or non-centrosomal-directed growth (Piehl et al., 2004). Indeed, potential types of MT assembly independent of the well-known models (Margolis and Wilson, 1978; Mitchison and Kirschner, 1984; Piehl et al., 2004) may also occur. In mature neurons, MTs can nucleate randomly throughout the whole cell (Stiess et al., 2010), which could be the source of the sophisticated morphologies found in neurons that may be related to some brain-specific functions, such as consciousness.

Indeed, the number and arrangement of MTs and how closely they extend to a specific zone may play critical roles in synaptic vesicle delivery and, thus, in signal transmission. The trillions of synapses could differ not only in shape and synapse area but in a multitude of differences in MT arrangement that could be responsible for the altered vesicular arrangement in Alzheimer's disease (AD) (Wang et al., 2023). Furthermore, the reduced MT density due to aging and AD may fundamentally change consciousness as we age and explain consciousness decline in these conditions (Cash et al., 2003; Zhang et al., 2015).

## Consciousness

Consciousness could be defined as the state of being aware of something (an environment) within oneself. However, other theories indicate that consciousness and awareness are different concepts since consciousness involves several stages, such as perceiving, feeling, and thinking, and those stages may require memory activity (Searle, 2000). Furthermore, consciousness may develop a memory system to create plans for the future (Budson et al., 2022), which is related to decision-making and planning (Budson et al., 2022). These definitions of consciousness may facilitate the search for its mechanisms based on biological and physical bases, including the most prominent theories of consciousness: higher-order landscape, global workspace, re-entry and predictive processing, integrated information, and other emerging theories (Seth and Bayne, 2022; Lenharo, 2024).

Regarding consciousness and brain localization, regions such as the thalamus or claustrum (Crick and Koch, 2003) connecting several cortical and subcortical areas could be involved.

Faster MT vibrations (Hameroff, 2012) could be a possible source of the observed EEG activity in consciousness that is found as a sequence of discrete events in synchrony with γ EEG, although this point has been discussed. It is suggested that there are 40 consciousness moments per second, related to fractal-like patterns of MTs (Hameroff and Penrose, 2014). These 40-Hz (γ waves) consciousness moments could be located in some cortex regions (Hameroff and Penrose, 2014) and are related to a very fast MT assembly–disassembly dynamic. Furthermore, it has been reported that MTs have inside the cell endogenous frequency oscillations in the range of 100 Hz (“high γ”) EGG (Cantero and Cantiello, 2020), in the other interneuronal γ connections (Singh et al., 2021). Furthermore, EEG γ waves may not be generated by axonal firing but by dendritic and soma interneuronal connections, suggesting that consciousness may be related to those changes in MTs present in dendrites and cell somas (Hameroff, 2010).

Indeed, in neurodegenerative disorders such as AD, resulting in progressive awareness (consciousness), the dynamics features of MT assembly-disassembly also are decreased (Peris et al., 2022), together with a decrease in γ waves (Mably and Colgin, 2018). Thus, the possible correlation of MT assembly dynamics, γ waves, and lack of consciousness could be compatible with the proposed Penrose's hypothesis.

## The entangled stage

The entangled stage could be defined as an ensemble of particles that cannot be described through individual particles but as a set. The ensemble is the result of the entanglement of two or more components, even if they are separated in space. The entanglement could occur through qubits. A qubit is a subatomic particle, like the spins of electrons or the spins of nuclear components such as protons or neutrons. The spin of all of those fermions (electrons, protons, or neutrons) could contain, in individual particles, a positive or negative charge. Upon entanglement of two of those particles with different charges, the result is a null charge. Spin changes could be used to look at an entangled stage. Furthermore, qubit is the basic unit in quantum computing, showing two relevant features: superposition and entanglement (Horodecki et al., 2009).

## Delocalized π electrons

Electrons are moving around the nucleus of an atom in different orbitals located at different distances of the nucleus, with π electrons present in π orbital. As previously described, π electrons have spin configurations (Fang et al., 1995) that could act as qubits. However, spins of π electrons are difficult to measure since they can entangle with the surrounding wet environment, causing de-phasing of any putative quantum coherent phenomena. However, an exception was suggested for a subatomic particle: the nuclear spin in phosphate atoms (Fisher, 2015).

Thus, we have a subatomic level with π electrons and atomic nuclei. These elementary particles have intrinsic quantum properties, for example, they have their spins. It was described that spin is the intrinsic angular momentum associated with these particles (Uhlenbeck and Goudsmit, 1925). For example, spin-up or spin-down states of these subatomic particles could be present, and quantum bits (qubits) can exist in both states simultaneously, permitting simultaneous answers to the computation they encode.

Recently, there has been a dawn of quantum biology in different biological processes (Ball, 2011). Although traditionally nuclear spin was not considered to play a role in biological processes, this view has changed more recently (Vardi et al., 2023). For brain studies, it has been proposed that only elements with a nuclear spin *I* = 1/2 (traditionally labeled like spin up and spin down) should be used (Fisher, 2015), with phosphorus nucleus being the only brain element with that particular spin (Fisher, 2015), a putative qubit.^1^ We will discuss below that GTP/GDP molecules are involved in MT assembly/disassembly. GTP/GDP is composed of guanine, ribose, and phosphates. Guanine contains 10 π electrons and phosphates have their nuclear spin (Figure 1). Nevertheless, there are some difficulties in using phosphorus (nuclear spin) as a suitable qubit transporter when memory storage is required. Phosphate ion (as qubit transporter) spreads out ~10 μm in 10^−2^ s (Nicholson and Sykova, 1998), but for qubit memory storage measurements, it may require times of seconds (or longer ones) as indicated by Fisher (2015).

## Present in the interior of brain microtubules

In Penrose's hypothesis, a role of the interior of MTs was proposed. Notably, in the interior of MTs, not only tubulin is present. Furthermore, brain MTs associated with Tau protein are located (Kar et al., 2003), independent of its presence in the outer surface (Ackmann et al., 2000). In addition, Tau protein could be modified by phosphorylation (Hanger et al., 2009) and the phosphorus (nuclear spin) of modified hyperphosphorylated-Tau may also play a role. Considering the role of Tau in consciousness, a recent comment has been published (Kosik, 2023). In addition to Tau, the phosphorylated neuronal β-tubulin subunit could play a role in consciousness (Diaz-Nido et al., 1990) (Figure 1).

## Discussion

The proposed role of the MT in consciousness–unconsciousness could take place in other events, such as anesthesia (see below). In unconsciousness or anesthesia, γ waves are missing, and δ waves are present (Frohlich et al., 2021). Inter- or intra-cellular wave changes may take place in processes such as unconsciousness, reversible coma, or sleep, and there are some similarities and differences among those processes. Among similarities, there is a presence in those processes of δ waves but not of γ waves (Frohlich et al., 2021). In addition, in a model of unconsciousness, such as propofol-induced anesthesia (Hameroff, 2021), δ waves are present (Frohlich et al., 2021). On the other hand, it could be possible to be awake and unconscious. As previously indicated, there is a neurological disorder, AD, that has been considered as a disorder of consciousness (Salmon et al., 2005; Huntley et al., 2021) found in awake persons. Indeed, a characteristic of unconsciousness, such as anosognosia, can be present in some AD patients in advanced stages (Prigatano, 2009).

Regarding the possible relation between changes in consciousness and intraneuronal changes, it was suggested, as indicated, that MTs may play a role at the cellular-molecular-quantum level in the consciousness process (Penrose, 2001). It was described that neuron MTs could form functional assemblies with specific frequencies (Frohlich et al., 2021) that can be regulated by neuronal brain MAPs such as tau protein. Tau protein's role in consciousness disorders, such as AD, can be analyzed in AD mouse models or in anesthetized mouse models. A correlation between tau modifications and anesthesia has been described by Chen et al. (2023). Propofol-induced anesthesia may activate protein kinase-like GSK3β (Huang et al., 2016), also known as tau kinase I, and the kinase will modify tau protein at specific residues that are found in AD (Hanger et al., 2009), preventing the normal assembly of MTs. Thus, tau protein may play a role in consciousness (see also Kosik, 2023).

In addition, looking at the effect of phosphor-tau in a transgenic mouse model overexpressing GSK3β, some features related to unconsciousness were found (Debski, 1976; Engel et al., 2006; Hooper et al., 2007). These features could be reversed by decreasing the level of phosphorylated tau (Llorens-Martin et al., 2013), and those mouse models could probably be used for further analysis of consciousness–unconsciousness transitions in both directions.

In conclusion, the influence of brain MTs on consciousness can be analyzed at different levels: (a) at the cellular (neuronal) level, where MT dynamics is regulated by GTP, and/or by the presence of MAPs, such as Tau protein; (b) at molecular level, exploring the role of GTP hydrolysis and the GTP/GDP binding to tubulin (the main component of MTs); (c) at molecular-atomic level, deciphering the role of kinases and phosphates from GTP/GDP bound to tubulin; and (d) at the subatomic level, by the proposed roles of π electrons and phosphorus spin nucleus as qubit transporters. The first three conclusions have been or could be further analyzed, but the main difficulty at present is analyzing the subatomic level. However, this is the main point for testing Penrose's hypothesis. A proposal that should be experimentally improved (or forgotten?) using innovative multidisciplinary approaches and novel instrumentation is available.

## Author contributions

JA: Conceptualization, Funding acquisition, Writing—original draft, Writing—review & editing. JM: Conceptualization, Writing—review & editing. GP-V: Conceptualization, Funding acquisition, Visualization, Writing—review & editing. VB: Conceptualization, Writing—review & editing. GP: Conceptualization, Funding acquisition, Writing—review & editing.

## Footnotes

^1^A qubit is the basic unit in quantum computing, showing two relevant features; superposition and entanglement (Horodecki et al., 2009).
